# Understanding the pharmacokinetics of Coartem^®^

**DOI:** 10.1186/1475-2875-8-S1-S4

**Published:** 2009-10-12

**Authors:** Abdoulaye Djimdé, Gilbert Lefèvre

**Affiliations:** 1 Malaria Research and Training Center, Department of Epidemiology of Parasitic Diseases, Faculty of Medicine, Pharmacy and Odonto-Stomatology, University of Bamako, P.O. Box: 1805 Point G, Bamako, Mali; 2 Translational Sciences, Novartis NIBR, Lichtstrasse 35, Basel CH-4002, Switzerland

## Abstract

Artemether and lumefantrine (AL), the active constituents of Coartem^® ^exhibit complementary pharmacokinetic profiles. Artemether is absorbed quickly; peak concentrations of artemether and its main active metabolite, dihydroartemisinin (DHA) occur at approximately two hours post-dose, leading to a rapid reduction in asexual parasite mass and a prompt resolution of symptoms. Lumefantrine is absorbed and cleared more slowly (terminal elimination half-life 3-4 days in malaria patients), and accumulates with successive doses, acting to prevent recrudescence by destroying any residual parasites that remain after artemether and DHA have been cleared from the body. Food intake significantly enhances the bioavailability of both artemether and lumefantrine, an effect which is more apparent for the highly lipophilic lumefantrine. However, a meal with only a small amount of fat (1.6 g) is considered sufficient to achieve adequate exposure to lumefantrine. The pharmacokinetics of artemether or lumefantrine are similar in children, when dosed according to their body weight, compared with adults. No randomized study has compared the pharmacokinetics of either agent in pregnant versus non-pregnant women. Studies in healthy volunteers and in children with malaria have confirmed that the pharmacokinetic characteristics of crushed standard AL tablets and the newly-developed Coartem^® ^Dispersible tablet formulation are similar. Studies to date in healthy volunteers have not identified any clinically relevant drug-drug interactions; data relating to concomitant administration of HIV therapies are limited. While dose-response analyses are difficult to undertake because of the low rate of treatment failures under AL, it appears that artemether and DHA exposure impact on parasite clearance time while lumefantrine exposure is associated with cure rate, consistent with their respective modes of action. In conclusion, knowledge of the pharmacokinetic profiles of artemether and lumefantrine is increasing within a range of settings, including infants and children. However, additional data would be warranted to better characterize artemether and lumefantrine pharmacokinetics in patients with hepatic impairment, in pregnant women, and in patients undergoing HIV/AIDS chemotherapy.

## The complementary pharmacokinetics of artemether and lumefantrine

Artemether and lumefantrine differ markedly in terms of rate of absorption and elimination. When administered as an oral combination, these differences act in a complementary manner to underpin the efficacy of Coartem^® ^(artemether/lumefantrine, AL) therapy. Artemether is absorbed rapidly, reaching a peak concentration at approximately two hours after dosing [1,2]. It is quickly hydrolysed to its main active metabolite, dihydroartemisinin (DHA), which also shows a maximum concentration within two to three hours after dosing [1]. Both artemether and DHA are very active anti-malarial agents that produce a reduction in asexual parasite mass of approximately 10,000-fold per reproductive cycle [3], accompanied by a prompt resolution of symptoms such as fever. In contrast, the lumefantrine component of AL is absorbed and cleared more slowly, acting to eliminate the residual parasites that may remain after artemether and DHA have been cleared from the body and thus prevent recrudescence [1,4]. Each tablet of AL (Coartem^®^) contains 20 mg of artemether and 120 mg of lumefantrine and the standard regimen consists of twice-daily administration for three days (six doses in total), covering at least two asexual parasite life cycles with artemether and optimising exposure to lumefantrine to prevent recrudescence. Twice-daily dosing maintains artemether and DHA concentrations at supratherapeutic levels [4] and a standard six-dose, three-day regimen of AL is estimated to reduce the parasite biomass by a factor of 10^8 ^[4].

A summary of pharmacokinetic parameters for artemether, DHA and lumefantrine obtained in a series of studies in healthy volunteers and malaria patients is presented in Table 1. The time to peak plasma concentration of artemether and DHA is similar in healthy volunteers [1] and malaria patients [5,6], but the peak level may be higher in individuals with malaria compared with healthy volunteers, possibly related to differences in the volume of distribution and intestinal absorption in malaria patients and the role of food in increasing oral bioavailability [1]. Artemether and DHA exhibit considerable variation in plasma concentration profiles both between individuals and from dose to dose, largely accounted for by their low and variable bioavailability and the influence of food on absorption [1].

**Table 1:** Pharmacokinetic parameters of artemether, dihydroartemisinin (DHA) and lumefantrine (mean or mean ± SD) among healthy volunteers and patients with malaria receiving a six-dose, three-day regimen of AL

			**Pharmacokinetic parameters**					
			
			**Artemether**			**DHA**		
				
**Subjects (n)**	**Food or fat content (g)**	**Country/Area**	**C_max_** **(ng/mL)**	**AUC** **(μg·h/mL)**	**t_1/2_** **(h)**	**C_max_** **(ng/mL)**	**AUC** **(μg·h/mL)**	**t_1/2_** **(h)**
Healthy volunteers (n = 14) [22]	~65 g^a^	Europe	30.8 ± 25.4	61.4 ± 87.5	2.0 ± 1.2	84.5 ± 26.5	178 ± 71	1.2 ± 0.4
Malaria patients (n = 13) [6]	7 g^d^	Thailand	34.8	66.4	1.5	165	367	1.3
Malaria patients	Local	Thailand	66.2 ± 54.3	211 ± 109	2.2 ± 1.0	205 ± 102	604 ± 259	1.6 ± 0.4

			**Lumefantrine**					
			
**Subjects (n)**	**Food or fat content (g)**	**Country/Area**	**C_max_** **(μg/mL)**	**AUC** **(μg·h/mL)**		**t_1/2_** **(day)**	**Day 7** **concentration (ng/mL)**	

Healthy volunteers (n = 14) [22]	~65 g^a^	Europe	10.0 ± 5.5	383 ± 304^e^		6.0 ± 1.3	-	
Malaria patients (n = 266) [7]	Local standard food	Thailand	9.0	561		3.2	-	
Malaria patients (n = 74) [13]	23 g^b^	Uganda	5.6 ± 2.7	-		-	460 ± 288	
Malaria patients (n = 17) [9]	6.4 g^c^	Thailand	7.0	410		4.1	350	
Malaria patients (n = 13) [6]	7 g^d^	Thailand	7.34	252^f^		2.83	384	

^a^FDA breakfast = 2 eggs, 2 strips bacon, 1 slice of toast with butter, 2 hash potatoes, 240 mL full-fat milk; ^b^10 g (300 mL milk) + 13 g (peanuts) or breast milk; ^c^200 mL carton chocolate milk; ^d^250 mL chocolate milk; ^e^AUC_62 h-last_; ^f^AUC_60 h-∞_

The peak concentration of lumefantrine occurs later, at approximately six hours post-dosing in healthy volunteers [1] and 3-4 hours in malaria patients [7]. Lumefantrine has an elimination half-life of approximately six days in healthy volunteers [8] and three to four days in patients with *Plasmodium falciparum *malaria [7,9] (Table 1). As observed with artemether, there is marked variability in the absorption of lumefantrine, particularly in patients with malaria, but this variation declines with successive doses [7] and lumefantrine bioavailability increases substantially [1]. Indeed, bioavailability has been estimated to increase three-fold for the third and fourth doses compared with the first and second doses during treatment with AL [2]. Febrile, nauseated patients tend to eat little during the early acute stages of malaria, and a return to normal food intake has been credited with improving lumefantrine bioavailability during treatment [1]. Because of its long elimination half-life, lumefantrine, as expected, naturally accumulates during multiple dosing [4], and the progressive increase in concentration observed during the course of therapy means that remaining parasites continue to be exposed to high levels of lumefantrine as artemether and DHA are cleared from the body.

## The influence of food

Food (especially dietary fat) enhances the bioavailability of artemether and lumefantrine, although this effect is more apparent for lumefantrine [1,7]. Administration of AL to healthy volunteers at the same time as a high-fat meal increases the bioavailability of artemether and lumefantrine by two-fold and 16-fold, respectively, compared with the fasted state [1]. This is particularly relevant in view of the low food intake by many patients during the acute phase of malaria. In a double-blind trial of 260 patients with uncomplicated malaria in Thailand, the extent and variability of lumefantrine absorption improved with clinical recovery as normal food intake was resumed [2]. The question arises how much dietary fat is required to achieve adequate lumefantrine exposure to achieve complete parasite clearance with AL. Ashley *et al *performed a cross-over pharmacokinetic study in which a single dose of AL was administered to 12 healthy volunteers in conjunction with different volumes of soya milk or with no milk [10]. A population model developed from lumefantrine concentration measurements demonstrated that 36 mL of soya milk (containing only 1.2 g of fat) was associated with 90% of the lumefantrine exposure obtained with 500 mL milk (16 g fat). The issue of adequate lumefantrine absorption has also been assessed in children with *P. falciparum *malaria in five African countries [11]. Data on relative lumefantrine exposure in 315 children receiving AL within a randomized trial were analysed according to concomitant consumption of different foodstuffs, or no food at all. The relative increase in mean lumefantrine absorption was 1.57 in patients drinking milk and 2.74 in those eating pancakes versus those who ate nothing, a much smaller difference than that seen between fasting and fed volunteers [1]. Interestingly, all of the 20 children with malaria who consumed no food at the time of any of the six AL doses in this study experienced parasitological cure. Premji *et al *have evaluated the typical fat content of African diets, weaning foods and breast milk [12]. They noted that total fat intake in most sub-Saharan countries is in the range 15-30 g/day during breast feeding, >10 g/day in the post-weaning phase, and 30-60 g/day for a normal diet, concluding that fat intake is typically adequate for optimal efficacy of lumefantrine. In a trial undertaken in Uganda in which 957 patients were randomized to receive AL either in hospital under supervision with a specified meal containing 23 g fat or unsupervised at home after the first dose with advice to take the drug with a meal or breast milk, both groups had an identical 28-day polymerase chain reaction (PCR)-corrected cure rate in evaluable patients (100%) [13]. A pharmacokinetic substudy revealed that lumefantrine plasma concentration was higher in the supervised group [13,14], but the identical efficacy results showed that typical food or milk consumption at home was sufficient to achieve adequate lumefantrine exposure for optimal efficacy. It appears that only a very small amount of dietary fat is necessary to ensure adequate lumefantrine absorption and that standard African diets or breast milk are sufficient to meet this need. It is important, however, that patients are encouraged to ensure normal food or milk intake during AL administration if possible and to resume intake quickly if food is declined during the acute phase of the disease.

## Pharmacokinetics of AL in specific patient populations

### Patients with renal or hepatic impairment

No specific pharmacokinetic studies have been performed in individuals with renal insufficiency. However, renal excretion plays no relevant role in AL clearance, as demonstrated by a randomized, open-label, single-dose trial involving 58 healthy volunteers, in which no quantifiable concentrations of artemether or lumefantrine were found in any urine sample [15]. Exposure to AL is, therefore, not expected to be modified in subjects with renal impairment compared to subjects with normal renal function. Furthermore, in clinical studies the adverse event profile of AL does not differ in patients with mild or moderate renal impairment compared with patients with normal renal function, although few patients with severe renal impairment took part in these studies [16]. Clinical evidence also indicates that renal status does not influences response rates or, indeed, that AL can affect kidney function. Bakshi *et al *undertook a retrospective analysis of 1,869 AL-treated patients, including 611 children ≥ 12 years [17], and found no significant changes in renal function following treatment with AL. Accordingly, no specific adjustment to AL dose is currently requested for patients with mild to moderate renal impairment, although caution should be exercized in patients with severe renal dysfunction [16]. Similarly, pharmacokinetic studies have not been carried out specifically in the setting of hepatic impairment. As with renal dysfunction, clinical studies have shown no difference in adverse events between patients with or without mild or moderate hepatic impairment [16] and the retrospective analysis of safety performed by Bakshi *et al *did not reveal any difference in adverse events in patients with hepatic insufficiency versus the general study population [17]. Dose adjustment is not required in patients with mildly or moderately restricted liver function, although a cautious approach is advised in the presence of severe hepatic impairment [16].

Overall, excellent tolerability has been observed in clinical trials over a wide range of AL systemic exposure. Taken together, the available data and the long post-marketing experience suggest that the use of AL may be safe in patients with some degree of renal or hepatic impairment.

### Paediatric patients

The greatest burden of malaria is borne by children, and understanding the pharmacokinetics of AL in this vulnerable group is critical. AL is indicated for use in malaria paediatric patients weighing as little as 5 kg (i.e. approximately two months of age), with dosing based on body weight: one tablet per dose if body weight is 5-<15 kg, two tablets per dose for 15-<25 kg, and three tablets per dose for 25-<35 kg. Two clinical trials in Africa have evaluated pharmacokinetic parameters in malaria paediatric patients given crushed AL tablets [18,19], which until the introduction of Coartem^® ^Dispersible was the standard method of drug administration in children. Results showed that exposure to artemether, DHA, and lumefantrine when dosed on a mg/kg body weight basis in paediatric malaria patients was comparable to that observed in adult malaria patients. Lumefantrine mean C_max _ranged between 4.71 and 9.37 μg/mL in paediatrics [18,19] and between 5.6 and 9.0 μg/mL in adult patients [7,9,13], while area under the curve (AUC)_0-last _values ranged between 372 μg·h/mL and 699 μg·h/mL in children [18,19], and between 252 μg·h/mL and 561 μg·h/mL in adults [6,7]. Artemether and DHA exposure in children [19] was also in line with that observed in adult malaria patients [5]. When artemether or DHA exposure was analysed according to body weight group, mean C_max _for artemether was 188 ng/mL, 198 ng/mL and 174 ng/mL in the 5-<15 kg, 15-<25 kg and 25-<35 kg groups, respectively (54.7 ng/mL, 79.8 ng/mL and 68.4 ng/mL for DHA). For lumefantrine, AUC_0-last _was 577 μg·h/mL in the 5-<15 kg body weight group, and 699 μg·h/mL in the 15-<25 kg group. Exposure in the highest body weight group (25-<35 kg) appeared to be higher (1150 μg·h/mL) but due to the small number of plasma concentrations collected in this group the finding was considered unreliable [19].

### Pregnancy

No randomized study has compared the pharmacokinetics of artemether, DHA or lumefantrine in pregnant and non-pregnant women. McGready and colleagues have reported population-derived lumefantrine plasma concentration time curves from 13 pregnant women in Thailand [6]. They compared the results with a historical group of 30 non-pregnant patients, most of whom were male. Their comparison showed that lumefantrine concentration curves were similar in the pregnant or non-pregnant groups during the first five days after treatment, after which total AUC (AUC_60-∞_) differed by only 6% (237 μg·h/mL in pregnant patients versus 251 μg·h/mL for the non-pregnant group). The peak lumefantrine concentration (7.34 μg/mL) was within the range of values reported in non-pregnant populations [7,9,13]. McGready *et al *observed that artemether and DHA C_max _and AUC_0-8 _values were lower in their 13 pregnant patients [6] versus another group of 25 male patients [5], but levels were similar to those seen in healthy volunteers [8]. Given the small patient population involved and the known inter-subject and inter-study variation in artemether pharmacokinetics, particularly if food intake differs, between-trial comparisons of drug exposure should be regarded with considerable caution. All 13 patients in the study by McGready *et al *achieved rapid cure with a median parasite clearance time of two days.

## Coartem^® ^Dispersible

**Figure 1:**
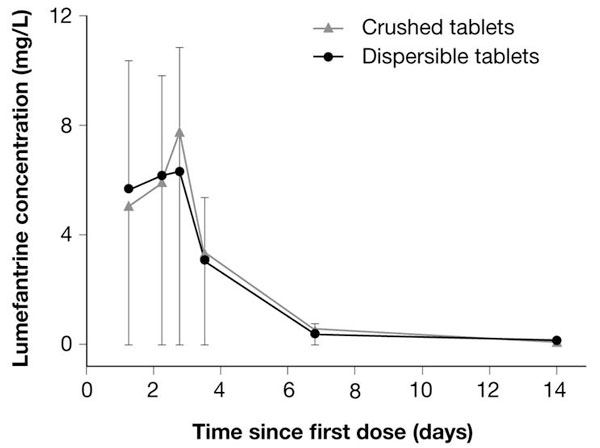
**Lumefantrine plasma concentration (mean ± SD) during treatment with dispersible or crushed standard tablets of AL in 625 children ≤ 12 years old (six doses over three days) **[19].

The new dispersible formulation of AL, developed specifically for use in children to provide improved ease of administration, has been evaluated in terms of its pharmacokinetic profile relative to crushed tablets of AL, the current standard of care in paediatrics. In an open-label crossover study, 48 healthy adult volunteers were given a single dose of the dispersible tablet or a crushed standard tablet under fed conditions [20]. Exposure to lumefantrine, artemether and DHA (AUC) was found to be bioequivalent for the dispersible formulation and the crushed standard tablet. Pharmacokinetic data from children with malaria receiving the dispersible tablet are available from a trial in five African countries in which children ≤ 12 years old were randomized to dispersible or crushed standard tablets [19]. In total, 184 patients provided blood samples for measurement of artemether and DHA concentration, and 625 patients provided one sample each for measurement of lumefantrine concentration. Mean artemether C_max _was 175 ± 168 ng/mL for patients randomized to dispersible tablets and 190 ± 168 ng/mL for those given crushed tablets. For DHA, these values were 64.7 ± 58.1 ng/mL and 63.7 ± 65.0 ng/mL, respectively. Pharmacokinetics were also assessed according to body weight group. Although no statistical comparisons are available no descriptive differences were apparent between body weight groups for either artemether or DHA [19]. No difference in lumefantrine pharmacokinetics was apparent between treatment groups (Figure 1). Mean peak concentration for lumefantrine over the six-dose treatment course was 6.3 ± 4.6 μg/mL in the dispersible arm and 7.7 ± 5.9 μg/mL in the crushed tablets arm, while mean AUC was 574 μg·h/mL and 638 μg·h/mL, respectively.

## Drug-drug interactions

Both artemether and lumefantrine are predominantly metabolized by the cytochrome P450 3A4 (CYP3A4) isoenzyme [1]. Although the likelihood of interactions between AL and other drugs is minimal in view of its short duration of administration and wide therapeutic index, three pharmacokinetic and pharmacodynamic drug-drug interaction studies with ketoconazole (a potent CYP3A4 inhibitor), mefloquine, and quinine have been conducted in healthy volunteers (Table 2). A randomized, open-label crossover trial in 16 healthy volunteers investigated the pharmacokinetics of artemether, DHA lumefantrine after a single dose of AL alone or in combination with multiple doses of ketoconazole [8]. There was a modest increase (2.4-fold) in artemether, DHA, and lumefantrine exposure but this was not associated with increased side effects or changes in electrocardiographic parameters, and AL dose adjustment is not considered necessary in falciparum malaria patients receiving ketoconazole or other potent CYP3A4 inhibitors. For mefloquine, oral administration prior to a standard six-dose regimen of AL in a randomized trial of 42 healthy volunteers had no statistically significant effect on plasma concentration of artemether or the ratio of artemether to DHA, but there was a 30-40% reduction in lumefantrine peak concentration and exposure (AUC) [21], possibly related to a mefloquine-induced decrease in bile production. The effect was not considered clinically relevant, and mefloquine pharmacokinetics were unaltered [21]. Potential pharmacokinetic interactions between AL and quinine have been assessed in a further randomized study of healthy volunteers given a two-hour intravenous infusion of quinine at the time of the last dose of AL in a six-dose regimen [22]. Neither the pharmacokinetics of lumefantrine nor the pharmacokinetics of quinine were influenced by the presence of the other drug. Plasma levels of artemether and DHA appeared to be lower following administration of quinine, but this was not considered to be clinically relevant.

**Table 2:** Effect of concomitant medication (ketoconazole, mefloquine, quinine or lopinavir/ritonavir) on exposure to artemether, dihydroartemisinin (DHA) and lumefantrine in studies undertaken in healthy volunteers

**Concomitant medication**	**N**	**Change in exposure to artemether (AUC)**	**Change in exposure to DHA (AUC)**	**Change in exposure to lumefantrine (AUC)**	**Change in exposure to concomitant medication (AUC)^a^**
Ketoconazole [8]	16	↑ 2.4-fold	↑ 1.7-fold	↑ 1.7-fold	Not measured
Mefloquine [21]	42	Unchanged	Unchanged	↓ 32%	Unchanged
Quinine [22]	42	↓ 46%	↓ 37%	Unchanged	Unchanged
Lopinavir/ritonavir [24]	13	↓ 35%	↓ 45%	↑ 2.4-fold	Unchanged

^a ^Ketoconazole, mefloquine, quinine or lopinavir/ritonavir, respectively

The increased anti-malarial failure rates that are observed in HIV-positive patients with malaria, due to an increased parasite burden and the reduced host immunity associated with HIV infection [23], mean that any drug-drug interactions in this population may be particularly relevant. Both anti-retroviral drugs and anti-malarial agents are metabolized through cytochrome P450 pathways, but due to variable pattern of inhibition and/or induction of cytochrome P450 enzymes, the role of anti-retrovirals in pharmacokinetic interactions is complex. To date, only limited data exist with respect to co-administration of AL and anti-retroviral drugs in either healthy volunteers or HIV-infected patients. A recent study in 13 healthy volunteers [24] showed that coadministration of the standard six-dose regimen of AL and lopinavir/ritonavir (400/100 mg twice daily), resulted in a significant 2.4-fold increase in lumefantrine exposure (AUC) (p < 0.01), a significant decrease in DHA exposure (p < 0.02) and in a non significant trend towards a decrease in artemether exposure (p > 0.05). However, there were no changes in the DHA:artemether AUC ratios and AL did not affect the pharmacokinetics of lopinavir or ritonavir. The observed increase in lumefantrine exposure is not surprising because ritonavir is a potent mechanism-based inhibitor of CYP3A4, and this finding is in agreement with data obtained when ketoconazole (another potent CYP3A4 inhibitor) is coadministered with AL [8]. The safety profile of AL, combined with the fact that lumefantrine AUC is a key predictor of parasitological cure, suggests that the observed increase in lumefantrine AUC in the presence of lopinavir/ritonavir may be beneficial in the treatment of malaria. The effects of ritonavir on drug-metabolizing enzymes are varied and complex. Although traditionally noted for its inhibitory effects on CYP3A and CYP2D6, ritonavir is also an inducer of the CYP enzymes 3A, 1A2, 2B6, 2C9, and 2C19, P-gp (mixed induction/inhibition) and uridine diphosphoglucuronyltransferase (UGT) [25]. The decrease in artemether and DHA exposure could have been due to the inducing effect of ritonavir on these cytochrome P450 enzymes, and/or UGT1A1 and UGT2B7, enzymes that are also involved in the metabolism of artemether to DHA and/or conversion of DHA to inactive metabolites [24]. Drug-drug interactions in HIV-positive malaria patients between AL and other anti-retrovirals or drugs used to treat HIV infection are currently being further evaluated.

## Dose-response relationships

The high response rates associated with the standard 6-dose regimen of AL limits the scope to evaluate the exposure-efficacy relationship because so few treatment failures occur. However, Ezzet *et al *have reported data from a study in which 260 patients with *P. falciparum *malaria or mixed infection including *P. falciparum *received only four doses of AL [2]. The derived pharmacokinetic data for artemether, DHA and lumefantrine were correlated with the 28-day PCR-corrected parasitological cure rate and the time to parasite clearance, and results showed that higher artemether and DHA AUC values decreased parasite clearance time (p < 0.001 for artemether and p = 0.003 for DHA) but were not associated with cure rate. In contrast, higher lumefantrine AUC values significantly increased the chance of cure but did not influence the time to parasite clearance. These findings are consistent with the mode of action of AL i.e. parasite clearance within the first 48 hours of treatment is largely due to artemether and DHA, while the long-lasting effect of lumefantrine acts to clear infection and prevent recrudescence. In their analysis, Ezzet *et al *identified the median lumefantrine concentration at day 7 to be 280 ng/mL, and found that the incidence of 28-day PCR-corrected cure was higher in patients with a day 7 level above this threshold [2]. In a subsequent study, Ezzet *et al *performed a pharmacodynamic analysis of lumefantrine in 266 patients with uncomplicated falciparum malaria receiving one of three different regimens of AL (four doses over two days, a standard six doses over three days, or six doses over five days) in a double-blind study [7]. Their findings confirmed that the longer duration of exposure to lumefantrine in the three- and five-day regimens was associated with higher 28-day PCR-corrected parasitological cure rates (83%, 97% and 99%, respectively; p < 0.01). The mean time for lumefantrine plasma concentration to decline to 280 ng/mL was 10.5 days with the standard three-day regimen. Pharmacokinetic data collected during a prospective, randomized study of AL in Uganda are consistent with these findings [14]. Among 479 adult and paediatric patients for whom lumefantrine data were available, median lumefantrine concentration at day 3 and day 7 in all three age groups (<5 years, 5-14 years and ≥ 15 years) was significantly lower in the intervention arm (unsupervised dosing) than the control arm (supervised dosing). Although the lumefantrine concentration at day 7 was more frequently below 280 ng/mL in the intervention (unsupervised) arm (58.8%), a fairly high proportion of the supervised patients (29.0%) also had concentrations below this threshold. Nevertheless, across the total study population the 28-day PCR-corrected cure rate in evaluable patients was 100% in both arms i.e. all patients achieved cure with no recrudescence [13]. White *et al *[26] recommend the routine measurement of drug concentration at day 7 as part of anti-malarial treatment. However, a day 7 lumefantrine concentration threshold of 280 ng/mL as a pharmacokinetic predictor for treatment response may not be applicable to all regions [13]. Indeed, more recently, a significantly lower threshold (175 ng/mL) was proposed by Price *et al *[27] based on a similar meta-analysis. Prediction of treatment failure showed a sensitivity of only 75% and a specificity of 84%.

## Conclusion

The pharmacokinetics of artemether and lumefantrine allow the two agents to act synergistically, achieving quick symptomatic relief with a high parasitological cure rate. Artemether has a rapid onset of action that reduces parasite biomass fast and resolves clinical symptoms. Indeed patients feel better faster with artemisinin-derived compounds than with any other class of anti-malarial drug. Lumefantrine acts longer-term to eradicate the remaining parasites over subsequent life cycles. The three-day course of AL reduces the parasite burden to approximately 10-10^4^parasites, a residual pool which is exposed to a high concentration of lumefantrine which has accumulated over successive doses. Moreover, the rapid parasite clearance achieved by artemether and DHA means that despite the short elimination half-lives of artemether and DHA, relatively few parasites are exposed to lumefantrine alone (and none are exposed to artemether or DHA alone), which would tend to reduce selective pressure for development of resistance. The new dispersible formulation shares these pharmacokinetic characteristics.

While food intake enhances absorption, particularly for lumefantrine, only a very small amount of fat is required to achieve adequate absorption, and typical African diets or breast milk are sufficient. Lastly, drug-drug interaction studies to date have not revealed any clinically significant interactions; trials with frequently-administered HIV therapies are ongoing.

In conclusion, knowledge of the pharmacokinetic profiles of artemether and lumefantrine is increasing within a range of settings, including infants and children. However, additional data would be warranted to better characterize artemether and lumefantrine pharmacokinetics in patients with hepatic impairment, in pregnant women, and in patients undergoing HIV/AIDS chemotherapy.

## Competing interests

The authors would like to acknowledge that Novartis Pharma AG sponsored this supplement. GL is an employee of Novartis Pharma AG; AD does not work for, or represent in any way, Novartis Pharma AG.

## Authors' contributions

All authors met International Committee of Medical Journal Editors criteria for authorship.
